# Remote Sensing Techniques for Water Quality Monitoring: A Review

**DOI:** 10.3390/s24248041

**Published:** 2024-12-17

**Authors:** Swapna A. Jaywant, Khalid Mahmood Arif

**Affiliations:** 1 Department of Mechanical and Electrical Engineering, Massey University, Auckland 0632, New Zealand; sjaywant@hauhau.co.nz; 2 HauHau Research, Auckland 0632, New Zealand

**Keywords:** remote sensing, water monitoring, space-borne sensors, airborne sensors

## Abstract

Freshwater resources are facing increasing challenges to water quality, due to factors such as population growth, human activities, climate change, and various human-made pressures. While on-site methods, as specified in the USGS water quality sampling handbook, are usually precise, they require more time, are costly, and provide data at specific points, which lacks the essential comprehensive geographic and temporal detail for water body assessment and management. Hence, conventional on-site monitoring methods are unable to provide a complete representation of freshwater systems. To address concerns regarding geographic and time-based coverage, remote sensing has developed into an effective solution, taking advantage of recent advancements in sensor technology and methodologies. The combination of GPS and GIS technologies, along with remotely sensed data, provides an efficient resource for continual monitoring and evaluation of water bodies. The use of remotely sensed data helps to establish a reliable geospatial database, serving as a standard for subsequent evaluations. The review emphasizes the contribution of remote sensing to water monitoring. It starts by exploring various space-borne and airborne sensors utilized for this purpose. Subsequently, the review explores remote sensing applications for water quality. Lastly, the review discusses the overall benefits and challenges related to remote sensing in water monitoring.

## 1. Introduction

Freshwater resources are crucial for sustaining ecosystems, encouraging human activities, and driving economic development on both regional and global levels. Freshwater ecosystems serve as habitats for various plant and animal species. These ecosystems include rivers, lakes, wetlands, and streams, each hosting unique flora and fauna. By providing suitable environments, freshwater bodies contribute significantly to the preservation of biodiversity. Also, freshwater ecosystems facilitate nutrient cycling by filtering and recycling organic matter, which is crucial for sustaining life within aquatic habitats. Numerous countries benefit from export revenues generated by industries reliant on freshwater resources. Agricultural commodities, such as crops and livestock, constitute a substantial portion of export earnings. Moreover, sectors like horticulture, viticulture (grape cultivation), forestry, fishing, and mining depend on freshwater inputs, contributing to export revenues through international sales of their products. Additionally, freshwater bodies support secondary sectors like tourism, recreation, and power generation, thus enhancing overall economic prosperity [1,2,3].

Despite their significance, freshwater resources are facing mounting water quality challenges driven by population growth, human activities, climate change, and various anthropogenic stressors [4,5,6,7]. For example, the application of fertilizers to boost agricultural yield is harmfully influencing water quality, detrimentally impacting both oceanic and aquatic ecosystems. Similarly, the escalating industrial water usage, particularly in developing nations where regulations for industrial wastewater management and water reclamation are lacking, is raising concerns regarding water quality maintenance and the reduction of water-related health issues [8]. The rise in temperature and subsequent polar ice melting is predicted to significantly elevate sea levels, leading to saltwater infiltration into groundwater aquifers. This renders groundwater unusable, consequently diminishing its availability [9]. Addressing these challenges requires the execution of comprehensive water management strategies, the implementation of sustainable practices, and concerted efforts to mitigate and adapt to environmental impacts. The resilience and health of water systems are paramount for safeguarding freshwater resources and the well-being of ecosystems and human societies alike. Therefore, effective management and conservation of freshwater ecosystems are essential for ensuring their long-term sustainability and fostering prosperity for future generations [1,10,11,12].

Managing water supplies amid ongoing worldwide changes needs continuously assessing water resources and susceptibilities across various locations and periods [8]. Usually, evaluating environmental stress in water bodies involves assessing water quality conditions. Additionally, it has relied on measuring ecological health by integrating laboratory and in situ analyses [13]. The conventional method of monitoring and assessing water quality is dependent on field measurements, sample acquisition, and laboratory examinations. These methods evaluate metrics associated with the physical, compositional, and ecological characteristics of the water body. However, in recent years, government bodies such as the United States Geological Survey (USGS) and the United States Environmental Protection Agency (USEPA), in collaboration with additional stakeholders, have developed sensor networks that offer ongoing water quality information almost instantly [14]. While on-site methods, like those described in the USGS water quality monitoring manual, are usually precise, they are nonetheless time-consuming, and costly, and provide location-specific data without spatial and temporal depth, which is essential for thorough evaluation of aquatic systems [15].

**Figure 1:**
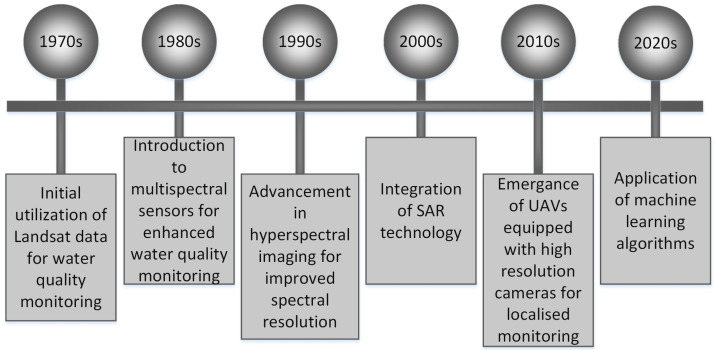
Timeline related to remote sensing techniques in water monitoring.

Thus, these challenges associated with comprehensive sampling present a major obstacle to effective monitoring and water quality management. As a result of these constraints, conventional on-site monitoring methods fall short of complete representation of freshwater systems. Remote sensing is considered an optimal approach to address problems concerning spatial and temporal coverage, utilizing the advancements in sensor technology and methodologies. This approach offers significant advantages, including extensive coverage and rapid update cycles, making it highly effective for monitoring and managing various environmental issues [16,17]. As space science progresses and computer applications with enhanced computing capabilities become more common, such methods have become essential tools for achieving this objective. These methods facilitate the optimal and efficient observation and identification of extensive areas and water bodies experiencing quality issues. Since the 1970s, remote sensing methods have continued to be extensively employed in modern water quality analysis efforts [18,19,20,21,22,23,24]. Remote sensing techniques for evaluating freshwater quality originated 50 years before the introduction of satellite technology. Figure 1 illustrates the timeline of these remote sensing techniques. Over this timeframe, numerous studies have showcased assuring remote sensing approaches for estimating the organic, chemical, and substantial properties of freshwater bodies [25]. Since the beginning of the 1970s, a broad spectrum of water quality components has been investigated by Landsat satellite sensors [19]. In the field of terrestrial remote sensing, algorithmic progress during the 1970s laid the groundwork for research publications concentrating on expansive and intricate spatial methods dating back to the mid-1980s [25]. These papers enclose studies on global land use [26], vegetation analysis at a global scale [27], and the relationships between primary productivity and carbon cycling [28]. By the late 1980s, the progression of ocean color remote sensing methods resulted in the creation of global datasets and estimates of oceanic primary productivity [29]. However, worldwide data resources for freshwater quality remain inadequate, with only a handful of notable instances, regardless of extensive recognition of their significance within the freshwater research community. This sluggish progression can be partly attributed to well-documented issues related to remote sensing of intricate aquatic systems and the lack of suitable sensors for monitoring freshwater quality. Since the early 2000s, procedural advancements have included the proven algorithm applications to identify spatial and temporal patterns, causes, and effects of varying freshwater quality on ecosystem dynamics and human communities [25]. According to Top et al. [25], the last ten years have seen significant advancements in inland water remote sensing, resulting in a deeper comprehension of inland water processes through the exploration of challenging scientific inquiries and the expansion of study scales. Currently, this effort persists with an expanding repository of accessible data, computing platforms, and techniques.

The review aimed to explore the contribution of remote sensing in water monitoring comprehensively. Initially, it extensively covers different space-borne and airborne sensors employed for this purpose, providing detailed discussions on their spectral, spatial, and temporal properties, which are conveniently presented in tabular format. Furthermore, the review offers a comparative analysis of these sensors, highlighting their respective advantages and limitations. The following section explores the various applications related to water quality monitoring and assessment. It provides thorough explanations of techniques used for observing optically responsive and non-responsive water pollutants, to assess water quality. Furthermore, the review emphasizes the primary benefits and challenges associated with remote sensing in water monitoring, offering valuable insights into the complexities of the field and its potential advancements.

## 2. Sensors Used in Remote Sensing Water Quality Monitoring

In general, remote sensing is the method used to acquire information related to an object or phenomenon with a lack of direct physical interaction. It encompasses the scientific identification of terrestrial surface characteristics and the estimation of their geophysical and biological properties through interactions with electromagnetic radiation. This process relies on sensing technology installed on drones, aircraft, satellites, or other platforms to gather information from a distance. The sensor/target possesses major characteristics such as spectral, spatial, temporal, and polarization signatures, which aid in target discrimination. Data from the Earth’s surface, captured by sensors in scattered, reflected, and/or emitted wavelengths, undergo radiometric and geometric correction prior to the retrieval of spectral data [30]. Within the domain of water quality monitoring, remote sensing is employed to determine radiation levels spanning the near-infrared and visible spectra, reflected off the water’s surface. This process captures the wavelengths at which water predominantly scatters and reflects incoming solar radiation, typically ranging from 400 to 900 nm. Solar radiation travels through the atmosphere before reaching the water body, where it undergoes modification due to absorption and scattering. As per Snell’s law, incident radiation reaching the water’s surface is transmitted or reflected from the surface and subsequently travels further into the water body [31]. These reflections or transmission can serve as direct or indirect means to measure various water quality metrics, including turbidity, total suspended solids (TSS), salinity, chlorophyll-a (Chl-a) concentration, Secchi disk depth (SDD), dissolved organic carbon (DOC), total phosphorus (TP), temperature, pH, and others. The spectral attributes of water and impurities, shaped by hydrological, biological, and chemical factors, among others, are crucial elements in water quality monitoring and assessment [18,32].

**Figure 2:**
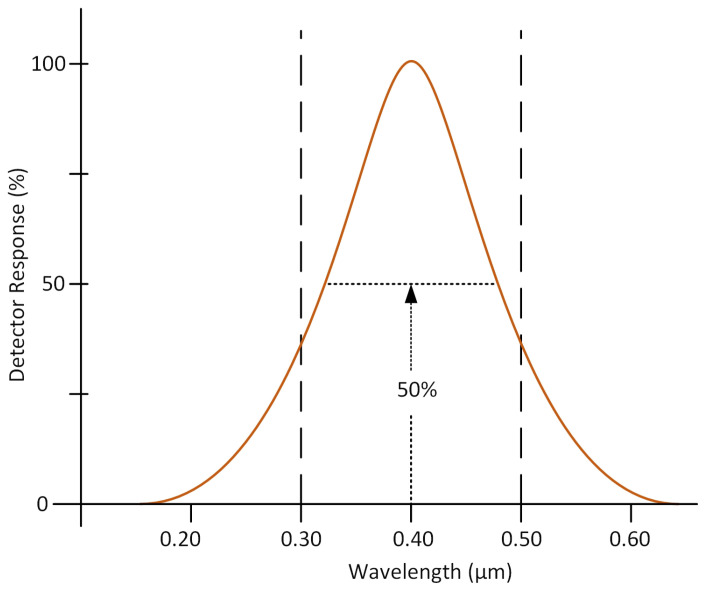
Illustration of spectral resolution. In this figure, the central wavelength is 0.40 μm.

Observing sensors are categorized into two primary types, depending on their platforms. Space-based sensors are deployed via spacecraft or satellites to positions beyond Earth’s atmosphere. Meanwhile, airborne sensors are installed on platforms in Earth’s airspace, such as balloons, aircraft, helicopters, boats, or Unmanned Aerial Vehicles (UAVs) [33]. These sensors play a crucial role in capturing images and related data while digitally recording the interaction of electromagnetic waves with matter. Image resolution is a vital characteristic of remote sensing, determining the ability to discern fine details within the image. Remote sensing has the following resolution types: spectral, spatial, radiometric, and temporal resolution. Spectral resolution is the wavelength interval captured by a detector, as shown in Figure 2. It is defined as the wavelength interval that is recorded at 50% of the peak response of the sensor. Spatial resolution pertains to the capability to differentiate between objects that are closely spaced in an image. Radiometric resolution represents the number of discrete levels, or bits, that an imaging system uses to record variations in a given range of values. Temporal resolution, for images captured of the same area, denotes the time interval between successive images [34].

### 2.1. Space-Borne Sensors

Space-borne sensors applied in water quality monitoring typically operate passively. These sensors offer extensive and periodic scanning of the Earth’s surface, ranging from daily to monthly revisits due to the consistent orbital patterns of satellites. This frequent coverage is particularly beneficial for studies requiring regular monitoring of water quality trends in specific areas. Further, space-borne sensors have been classified into two types depending on the count of spectral bands they use. These types are multispectral sensors and hyperspectral sensors [33].

Multispectral sensors gather data across a defined number of distinct spectral bands, typically spanning a few spectral bands, generally ranging from 3 to 10 bands. The images generated by these sensors are called multispectral images and represent the main type of imagery captured by remote sensing (RS) radiometers. Compared to panchromatic (black and white) images, multispectral images contain additional data; each pixel captures the total intensity of visible radiation from its corresponding section of the scene. Typically, satellites include the red–green–blue (RGB) region, which consists of three or more wavelength bands within the 0.4–0.7 μm range. Infrared wavelengths, spanning from 0.7 to 10 μm or beyond, are further categorized into near-infrared (NIR), middle-infrared (MIR), and far-infrared (FIR or thermal) groups. While multispectral sensors provide adequate spectral coverage for numerous applications, their limited number of bands may constrain the level of detail that can be captured compared to hyperspectral sensors [33].

On the other hand, hyperspectral sensors can capture data across hundreds or even thousands of adjacent spectral bands, with very narrow bandwidths. This high spectral resolution allows hyperspectral sensors to distinguish minute differences in the spectral signatures of various materials present in water bodies. By analyzing the unique spectral fingerprints of different substances, hyperspectral sensors can provide more detailed and accurate information about water quality parameters [33]. The choice of multispectral and hyperspectral sensors is determined by the particular requirements of the water quality monitoring application. Multispectral sensors are often favored for broad-scale assessments and monitoring of large water bodies, where general trends and patterns are of primary interest. Meanwhile, hyperspectral sensors are more suitable for applications requiring detailed analysis and identification of specific constituents or contaminants within water bodies, such as detecting oil spills or monitoring algal blooms. Table 1 provides a summary comparing hyperspectral and multispectral sensors.

Multispectral data are utilized for remote sensing water quality measurement. This dataset comprises OLI, TM, MSS, ETM+, ESA’s Sentinel-2, France’s SPOT satellite data, ENVISAT MERIS, and NOAA’s AVHRR data. However, datasets such as Landsat MSS are no longer accessible, and satellite missions like Envisat have concluded. In water quality monitoring, Landsat series data are primarily used, considering factors such as accessibility and resolution (spatial, temporal, and spectral). This information is particularly suitable for monitoring parameters including total suspended matter, TP, and chemical oxygen demand (COD). However, due to resolution limitations, multispectral models used in remote sensing for water quality assessment primarily rely on empirical methods, which are better suited for specific timeframes or water areas [35]. Hyperspectral satellites offer numerous bands with a spectral resolution of approximately 0.01 μm. Data from hyperspectral sensors, such as China’s HJ-1 satellite’s HIS and the USA’s Hyperion, have been utilized for water quality measurements [36]. The abundance of bands in higher spectral resolution data allows for precise and optimal selection, enhancing the differentiation of spectral mixing discrepancies in multispectral data. This significantly improves the accuracy of algorithms for retrieving water quality parameters and indicates promising potential for their application [35,37].

The data extractable from an image is determined with the spatial resolution of the sensor, denoting the smallest noticeable feature size from space. Thus, this study categorizes sensors into three types based on their pixel size and spatial resolution in generated images: low regional/global spatial resolution, moderate resolution, and high spatial resolution. Apart from their ability to detect water resources, high spatial resolution sensors can monitor waterways and small lakes. Commercially available satellite images vary in cost based on their spatial resolution and coverage. Higher resolution systems tend to be more expensive, while moderate resolution ones, such as Landsat, may be available at a lower cost or even for free. Additionally, global systems (i.e., Moderate Resolution Imaging Spectroradiometer (MODIS) and medium resolution Imaging Spectrometer (MERIS)) may offer images at various price points, depending on their resolution and coverage [33]. Table 2 lists commonly used space-borne sensors along with the spatial, spectral, and temporal resolutions of each.

There are various primary advantages of utilizing space-borne sensors for water quality monitoring. With frequent revisits ranging from daily to monthly intervals, space-borne sensors are invaluable for conducting multi-temporal studies and tracking trends and tendencies in water quality over time. Additionally, the high spatial resolutions offered by certain commercial sensors, such as the QuickBird, IKONOS, WorldView series, and SPOT-5/HRG, prove advantageous for studying small water bodies and implementing project-based evaluations. Moreover, moderate, regional, and global sensors capable of covering extensive geographic regions in each scene are well-suited for local water quality research. Furthermore, the utilization of space-borne sensors is often cost-effective, and in some cases, even free, making them particularly appropriate for widespread water quality remote sensing operations [38].

However, space-borne sensors used in water quality studies exhibit several typical shortcomings. Firstly, the spectral resolution of hyperspectral airborne sensors is generally more detailed than that of multispectral space-borne sensors. Additionally, a few space-borne sensors have limited coverage of the electromagnetic spectrum, lacking significant wavelengths like the blue, middle infrared, and thermal bands, which can impact the accuracy of water quality parameter estimation. Moreover, obtaining high spatial resolution imagery often incurs substantial costs, posing a financial constraint. Cloud cover also presents a significant challenge, complicating project schedules and making it difficult to remove cloud cover from images. Furthermore, employing empirical and semi-empirical methods to analyze multispectral imagery might result in inflated concentration levels for certain water quality parameters, like Chl-a in shallow waters, where bottom reflectance impacts the reflectance from the water surface [33,38].

### 2.2. Airborne Sensors

In general, airborne sensors offer higher spectral and spatial resolutions than their space-borne counterparts. Consequently, despite certain drawbacks like higher image costs, data volume, and processing expenses, airborne hyperspectral remote sensing methods may be seen as a more effective approach for assessing water quality variables. The resolution in airborne imagers is determined by factors such as duration of integration and the aircraft’s minimum speed [33]. Hyperspectral airborne data are commonly combined with on-site measurements to evaluate water quality. For example, in a study conducted by C. Cillero Castro et al. [39], eutrophication monitoring of small reservoirs was conducted using this method.

Hyperspectral airborne sensors are passive devices developed to gauge the reflectance of solar radiation across a broader array of spectral bands. These bands span both infrared and visible wavelengths of the electromagnetic spectrum. This extensive coverage, marked by adjacent and narrow bandwidths unique to hyperspectral data, enables intricate monitoring of surface features on Earth. These finer distinctions surpass the capabilities of multispectral scanners, which rely on comparatively coarser bandwidths, thereby revealing details otherwise undetectable. Similarly to space-borne sensors, it is essential to eliminate atmospheric interference from airborne sensors. This process enhances the image quality and image accuracy, thereby enabling adequate interpretation for water quality assessments. To accomplish this, precise atmospheric models are employed. These models use the sensor’s radiance data to calculate surface reflectance. The calculation converts radiance into reflectance. This process eliminates the atmospheric influences caused by sunlight traveling through the scene and reflecting back to the aircraft [33]. Numerous models for atmospheric correction procedures are available, typically implemented through image processing software like ITT Visual Information Solutions (ITT Vis) ENVI and ERDAS Imagine. These tools employ various correction methods, including empirical methods that necessitate field spectra for bright and dark targets, as well as atmospheric removal algorithms (such as ATREM) that do not require field data. ATREM algorithms, like ACORN, HATCH, ISDAS, ATCOR, and FLAASH, are frequently utilized for terrestrial applications. For oceanic applications, atmospheric correction techniques utilize radiative transfer equations, like Tafkaa developed by WATCOR and the Naval Research Laboratory designed for Shoreline waters and freshwater lakes [33,40]. Table 3 describes the specifications of commonly used air-space sensors in water quality monitoring.

Hyperspectral airborne data present distinct advantages for studying water quality parameters across various scales. Their configurational flexibility, including spatial resolution, number of bands, bandwidth, and spectral range, allows customization to achieve particular project objectives and budget limitations, and the consideration of measurable water quality parameters. With greater spatial and spectral resolutions, they are particularly suitable for assessing water quality on small scales, enabling evaluations in small water bodies, rivers, Streams, small lakes, and river mouths. However, challenges related to bottom reflectance need to be addressed. At higher altitudes, they can span broader regions, rendering them useful for local water quality investigations. Moreover, hyperspectral imaging can leverage spatial correlations between spectra within the area, facilitating the development of more advanced spectral-spatial models for precise image segmentation and classification [33,41].

Various ground-measured spectral data and airborne remote sensing data enhance the capabilities for remote sensing water quality assessment. As a result of the speedy progress in UAV technology, compact and lightweight UAV systems, featuring infrared sensors, multispectral cameras, high-resolution spectrometers, and LiDAR, have emerged as useful and efficient tools in water quality management [42]. However, like satellite-based multispectral sensors, most UAV sensors currently available are designed primarily for terrestrial applications, especially precision agriculture, and their spectral bands are not optimized for aquatic environments. However, their use in marine environments is rarely recorded. Despite this, they can still be effectively employed in certain water-related applications, such as quantifying the pigment Chl-a [39]. In complex water environments and narrow urban rivers, combining UAV remote sensing data with a limited number of on-site water quality samples can provide a comprehensive overview of water quality and spatial distribution. This approach enhances the efficiency and accuracy of monitoring, contributing to the effective protection and management of urban waterways [43]. Building on this background, a multi-sensor and multi-scale monitoring tool was developed for spatially explicit and periodic monitoring of eutrophication in a small drinking water reservoir [39]. Similarly, this technology shows great potential for monitoring water quality in coastal environments. A study was conducted to evaluate its ability to infer the spatial distribution of Chl-a concentration and turbidity in surface waters by exploring the potential of estimating these parameters using reflectance ratios and indices calculated from different wavelength band pairs [43].

Notably, instruments like the USA’s AVIRIS, featuring 220 channels, and Canada’s Compact Airborne Spectrographic Imager (CASI), equipped with 48 channels, have seen extensive use in water environment monitoring. Additionally, data from airborne Chinese Imaging Spectrometer (CIS) contribute to water environment monitoring efforts. However, airborne data collection faces challenges such as high flight costs and associated risks. Portable spectrometers offer versatility for onboard use, allowing for the detection of reflectance from water surfaces across adjustable wavelength ranges. Widely used field spectrometers like ASD Field Spec Pro, PSR-1100, and L1-1800 are instrumental in water environment management. Non-satellite spectrometers provide benefits such as superior spatial and spectral resolution, enabling continuous spectral characterization of ground features. Furthermore, non-satellite remote sensing data are more resistant to atmospheric interference compared to satellite data. Nonetheless, non-satellite data collection via aircraft or ground measurements is costlier and constrained in its ability to comprehensively observe large lake and river areas [35].

However, these sensors come with certain limitations. Airborne hyperspectral image processing is inherently more intricate and expensive than space-borne surveys due to the large and multidimensional nature of hyperspectral cubes, which can potentially exceed hundreds of megabytes in size. Planning for airborne surveys requires careful consideration of factors such as aviation traffic, sunlight exposure, atmospheric conditions, and flight path alignments. Additionally, airborne surveys are often costlier than space-borne sensors. Effective image processing demands advanced computing systems, precise detectors, and substantial storage potential. Furthermore, airborne sensors generally encompass smaller regions as a result of the lower altitude of image acquisition compared to space-borne sensors [33,38]. In Table 4, there is an overall comparison between space-borne and airborne sensors.

### 2.3. Other Sensors

Other sensor categories widely used in oceanic remote sensing include synthetic aperture radar (SAR) and microwave radiometers (MWR). Passive microwave radiometers detect energy emissions at frequencies spanning from 1 to 1000 GHz (across sub-mm to cm wavelengths), commonly referred to as microwaves. Ocean researchers are able to derive significant water quality constituents, such as sea surface salinity and sea surface temperature (SST). This is achieved through understanding the physical mechanisms related to energy emissions at these specific wavelengths [18].

Synthetic aperture radar (SAR) is a radar technology utilized for generating 3D images of objects, and it is suited for deployment on aircraft or spacecraft [18,44,45,46]. The primary advantage of SAR data are its ability to penetrate clouds and operate independently of sunlight, enabling rapid observation of large disaster-affected areas regardless of weather conditions or time of day. Despite SARs being extensively employed for tasks such as detecting water pollution, including oil spills, mapping ocean surface features, determining sea surface wind speed, and conducting geographic monitoring, their use in water environment studies and measurement of water quality variables is relatively infrequent. When SAR data are integrated with information from other sensors in water quality assessments, the findings have indicated that SARs provide only marginal assistance in enhancing the measurement of water quality variables, as exemplified in the study by Zhang [47].

There is potential to evaluate water quality parameters using the microwave wavelength of the spectrum, even though most studies have traditionally assessed these parameters using the near-infrared and visible portions of the electromagnetic spectrum. Microwave sensors offer distinct advantages over visible and near-infrared sensors, as they are minimally affected by atmospheric interference. This makes them well-suited for detecting various biological and physical attributes, including those related to vegetation, terrain, coastal areas, shoreline regions, and severe weather, regardless of the time of day or night [48,49]. Furthermore, microwave sensors deployed on aircraft have demonstrated the capability to estimate temperature distributions with a precision of approximately 1 °C. Similarly, microwave radiometers can be utilized to determine salinity distribution. Microwave remote sensing has also been investigated for its potential in estimating numerous water quality parameters, like turbidity, suspended sediment concentration, salinity, transparency, Chl-a concentration, and total dissolved solids [50].

The following section explores the application of these sensors in developing remote sensing-based techniques for water quality monitoring. It focuses on the methodologies, algorithms, and data integration strategies used to maximize sensor potential for accurately assessing and monitoring essential water quality parameters.

## 3. Remote Sensing Based Water Quality Monitoring Technique and Applications

**Figure 3:**
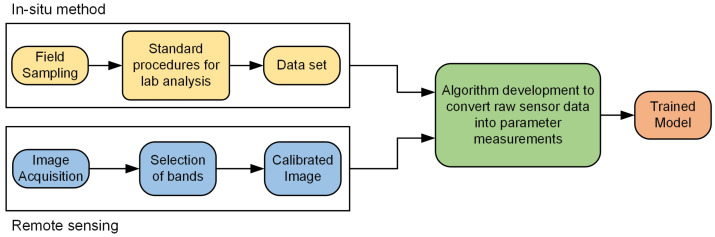
Stages involved in developing in-situ and remote sensing methods.

When developing a remote sensing technique for water monitoring (Figure 3), an essential first step entails gathering a comprehensive water dataset through in situ methods for assessing water quality. This dataset serves as the foundation for subsequent remote sensing analysis. Remote sensing can be conducted using either satellite-based platforms or by employing cameras mounted on UAVs, commonly referred to as drones. Once the dataset is acquired, it is combined with the necessary remote sensing data, such as spectral imagery or other relevant measurements. This combined dataset is then utilized for the application of algorithms aimed at extracting valuable information related to water quality parameters. The role of algorithms is essential here, as they evaluate remote sensing information and derive important data about water quality. Utilizing machine learning and deep learning techniques, scientists can develop sophisticated algorithms capable of accurately assessing water quality parameters from remote sensing data points. For example, Alnahit A. O. et al. [51] used Random Forest and Boosted regression tree models to predict water quality in selected watersheds. Najafzadeh and Niazmardi [52] introduced a multiple-kernel support vector regression algorithm, modifying standard support vector regression to estimate water quality parameters. Additionally, a nine-layer multilayer perceptron was used in conjunction with a K-nearest neighbor algorithm for water quality prediction in another study [53]. These algorithms facilitate the automation of analysis, making it possible to quickly and efficiently monitor water bodies across large areas [54,55]. These techniques are suitable for evaluating water quality.

The spectral properties of a clean water surface are considerably different from those of water with pollutants or contaminants. A clean water body reflects only 1–3% of incident radiation while absorbing approximately 97–99% of incoming energy [8]. This ratio changes based on water quality, given that contaminated water demonstrates increased reflectance. Moreover, the primary reflected wavelength shifts as the composition of water alters. Consequently, the occurrence of diverse compounds in the aquatic environment generates distinct spectral patterns, which are captured by many optical and thermal sensors installed on various airborne platforms and satellites. Developing a connection between water quality parameters and spectral reflectance is vital for monitoring water quality [8,56], with the typical representation of this relationship described by the following equation.

(1) Y=a−bX,

where *Y* represents the spectral reflectance, *X* represents the water quality parameter, and *a* and *b* are the empirical factors [8].

Water impurities are classified as optically active and inactive. Optically active constituents (OAC) of water comprise the portion of dissolved and suspended matter that interacts with light through absorption, refraction, and scattering processes. These interactions, including absorption, refraction, and scattering of light, are unique to every constituent and are collectively termed as the inherent optical properties (IOP) [57]. The reflectance (*R*) of light from a water surface depends on the refractive index of the medium, which is affected by factors like wavelength, temperature, and salinity [58]. Since OACs change with factors like temperature and salinity, they are classified according to their spectral water-leaving radiance into categories such as pure water, colored dissolved organic matter (CDOM), non-algal particles, and Chl-a (and other phytoplankton pigments) [58]. Chl-a belongs to the phytoplankton category of optically active constituents and is directly linked with algal matter and associated elements found in the water body.

Chlorophyll is essential for absorbing solar radiation in the aquatic column and is involved in photosynthesis during the light cycle of algae. Chlorophyll pigments, like Chl-a found in plants, algae, and cyanobacteria, are responsible for the green coloration observed in these organisms. Chl-a absorbs energy from all other wavelengths while reflecting only green. Its presence in water bodies is directly associated with algal bloom occurrences. It serves as an indicator of eutrophication levels. Eutrophication, a natural process, is accelerated by nutrient loading, particularly nitrogen and phosphorus from fertilizer leachate and fossil fuel combustion. These compounds stimulate algae growth, hastening the degradation of water bodies [8,58,59,60]. Variations in Chl-a levels in water generate spectral reflectance curves characterized by reflectance in the green (∼0.5 μm) and near-infrared (∼0.8 μm) bands, and absorption in the blue (∼0.4 μm) and red (∼0.7 μm) bands. Different sensors leverage this feature to obtain Chl-a concentration from water. Though visible wavelengths from multispectral sensors are frequently used for Chl-a estimation in many studies [8], most of the literature suggests that the optimal bandwidths for quantifying Chl-a levels are near 675 nm and 700 nm [18]. Equation (1) outlines a comprehensive empirical model that establishes the correlation between spectral bands and water quality parameters. Monitoring Chl-a concentrations could see considerable enhancement through the use of multiple spectral bands [8]. Consequently, numerous studies have employed band ratios, effectively mitigating atmospheric effects and enhancing the signal-to-noise ratio [8,61]. The equation below was formulated to calculate Chl-a concentration through band ratios [8].

(2) $$\log_{10}\left[\mathrm{Chl}-a\right]=A+B\left(-\log_{10}G\right),$$

$$\begin{array}{cc} with\;G & =\frac{{\left(R_{2}\right)}^{2}}{R_{1}*R_{3}} \end{array}$$

where *A* and *B* represent constant resulting from on-site readings, and *R*_1_, *R*_2_, *R*_3_ are the spectral reflectance at 460 nm, 490 nm and 520 nm, respectively [8].

Moreover, various algorithms with multiple spectral bands have been developed such as the normalized difference chlorophyll index algorithm (NDCI), two-band algorithm (2BDA), three-band algorithm (3BDA), fluorescence line height (FLH) algorithm, maximum chlorophyll index (MCI) algorithm, normalized green-red difference index (NGRDI), and surface algal bloom index algorithm (SABI) [8].

The category of non-algal particles within the OACs includes suspended particulate matter ranging from 0.2 to 0.7 mm. This encompasses non-pigmented elements of phytoplankton, organic detritus, various living microorganisms like zooplankton and bacteria, as well as inorganic particles originating from riverbed erosion, runoff, and particle resuspension. Suspended sediments impact water quality by modifying nutrient levels, obstructing light transmission, reducing dissolved oxygen concentrations, and clogging channels. Their presence induces turbidity in the water, with turbidity directly correlating to suspended particle concentration—greater turbidity signifies higher suspended particle concentration. Thus, turbidity measurement is frequently regarded as a surrogate for sediment concentration in water bodies [8,62]. Suspended sediment and turbidity are also classified as non-algal particles, often leading to increased reflectance in the visible and near-infrared (NIR) bands. Consequently, these variables harm water column transparency and often serve as primary carriers for both nutrients and contaminants [31]. Various empirical models have been employed to demonstrate the relationship between the levels of suspended particles and spectral reflectance. For example, JA Harrington et al. [63] developed a model expressed by the following equation:

(3) $$R_{i}=B_{i}\left[1-e{(}^{c/S_{i}})\right],$$

In Equation (3) *R_i_*, represents the reflectance associated with band *i*, *c* denotes the concentration of TSS. *S_i_* and *B_i_* are statistically determined coefficients. *B_i_* signifies the reflectance saturation of band *i* at high total suspended solids (TSS) levels, and *S_i_* denotes the TSS concentration at 63% saturation reflectance of band *i* [8]. Due to the limited applicability and accuracy of empirical models across varying conditions or environments, more universally applicable and theoretically grounded models, known as Radiation Transfer Equations (RTEs), have been developed. Such RTE-based models assist in resolving common challenges encountered in empirical modeling, including (i) interference from bottom reflection in water bodies, (ii) inaccuracies in estimated retrievals, and (iii) precise determination of optical properties of water bodies [64].

CDOM is the primary measurable parameter in remote sensing, commonly employed as a measure of DOC and total organic carbon (TOC). The origin of DOC can be categorized as either autochthonous, which is derived from the decomposition of algae or hydrophyte within the surface water. Alternatively, it can be allochthonous, representing an external origin such as from soils or terrestrial plants [58]. An elevation in CDOM concentration influences the chemical, physical, and biological characteristics of water sources. Elevated CDOM concentrations result in light diminution within water bodies and promote phytoplankton growth, consequently enhancing water body eutrophication [65]. Hence, the existence of CDOM impacts the framework and operation of the river habitat. CDOM exhibits significant absorption within the ultraviolet (UV) and visible spectrum and is conventionally quantified by measuring its absorption at a wavelength of 440 nm [31]. This wavelength coincides with the absorption band of Chl-a, making it challenging to distinguish between CDOM levels and Chl-a concentration [8]. The utilization of hyperspectral remote sensing datasets to detect CDOM levels is increasingly significant due to the challenge of determining CDOM concentrations among suspended solids and chlorophyll content. A matrix inversion method was developed to extract CDOM levels from the EO-1 Hyperion hyperspectral dataset, which exhibited sufficient sensitivity to detect CDOM, Chl-a, and TSS concentrations in complex water environments [66]. Another approach for hyperspectral remote sensing inversion was proposed to determine the absorption coefficient for CDOM [67]. NC Tehrani et al. [68] utilized three datasets—SeaWiFS, MODIS, and MERIS—to estimate CDOM concentrations, and determined that the ratio of the 510 nm and 560 nm bands from the MERIS dataset yields the most accurate results. In a recent study, B. Juhls et al. [69] assessed the absorption coefficient of CDOM using the MERIS dataset in the Arctic shelf area. The suggested retrieval algorithm proved effective in waters with extreme absorption and high scattering, characterized by high optical complexity spanning the fluvial-marine transition.

Efforts to integrate on-site and satellite observations for comprehensive surveillance of shoreline regions have been significant over the last couple of decades. According to Arabi et al. [70], the combination of time-space water constituent concentration (WCC) data from on-site measurements and satellite imagery holds promise for anomaly detection. It functions as an early alert system for management measures in the intricate marine environments of the Wadden Sea. They utilized multi-sensor satellite imagery combined with on-site hyperspectral readings, employing Radiative Transfer modeling across the Wadden Sea of the Netherlands, to extract a 15-year daily cycle of WCCs. Their findings reveal a strong agreement between WCCs based on terrestrial remote sensing measurements (Rrs) at the water surface level and those obtained from satellite imagery at high altitudes, showing consistent temporal patterns from 2003 to 2018 at the NIOZ jetty location. However, two primary limitations affecting the organized or regular assessment of marine systems via satellite remote sensing are as follows: firstly, atmospheric effects can deprive water body managers of data for long durations, and secondly, the spatial resolution of sensors aboard satellites is limited. Multispectral sensors primarily intended for ocean water monitoring exacerbate these limitations. In light of these challenges, C. C. Castro et al. [39] propose a methodology for aquatic resource monitoring to maximize data collection periodicity and bridge gaps through integrating sensors and systems, thus enhancing existing monitoring programs. The tool utilizes open-access satellite and ground-based data in addition to UAV-based technology. They evaluated three different sensors for Chl-a retrieval, a quantitative indicator of phytoplankton biomass and ecosystem trophic state: the MultiSpectral Instrument, the Operational Land Imager, and the RedEdge Micasense. The results demonstrated a fairly strong concordance among these sensors across various spatial resolutions (10 m, 30 m, and 8 cm). This indicates a significant potential for developing a comprehensive and integrated monitoring strategy for assessing the trophic state of small water bodies. R. McEliece et al. [55] endeavoured to assess water quality using UAV multispectral sensors, leveraging technology originally intended for farming purposes and adapting methods from satellite remote sensing techniques. UAV multispectral sensors offer the capability to capture extensive information in coastal areas within one 20 min UAV flight. This investigation laid the groundwork for the potential creation of a robust tool for environmental scientists. However, numerous technological advancements are still required to fully realize the possible use of UAV multispectral sensors for water quality mapping. Addressing depth and bottom reflectance issues is crucial for developing water quality estimations with UAV multispectral imagery within nearshore environment [55].

Water pollution undergoes nonlinear regression influenced by numerous factors, thus limiting the effectiveness of water quality reconstruction outcomes when employing traditional linear inversion models [71]. As artificial intelligence has progressed, machine learning technologies have increasingly been applied in remote sensing. Given their ability to tackle complex nonlinear issues, machine learning algorithms like support vector regression (SVR) and partial least squares regression (PLSR) have been employed to determine water quality parameter concentrations [72,73,74,75]. Overall, machine learning demonstrates strong nonlinear approximation capabilities, offering a novel approach to enhance the precision of water quality monitoring. E. E. Alves et al. [76] used principal component analysis. They applied this method to enhance the input parameters for a feed-forward neural network. The modeling results identified the optimal ANN architecture as 19-16-1, with trainlm as the training function. The model achieved a root mean square error (RMSE) of 0.5813, a determination coefficient R^2^ of 0.9857 (*p* < 0.0001) between observed and predicted values, and a mean absolute percentage error (MAPE) of 0.57 ± 0.51%. Consequently, they achieved accurate inversion of the water quality index (WQI), and the findings demonstrated the potential of using a portable UV–Vis spectrophotometer connected to a computer for WQI prediction in areas lacking the infrastructure for conventional methods, as well as for real-time monitoring of water bodies. R. Gogu et al. [77] demonstrated the promising potential of employing a neural network for estimating river water salinity levels through their experiments. X. Wang et al. [71] assessed the WQI of the Ebinur Lake catchment area. They utilized the SVR model with near-surface spectroscopy. This study integrated a machine learning algorithm, WQI, and remote sensing spectral indices (difference index [DI], normalized difference index [NDI], and ratio index [RI]) using fractional derivatives methods to develop a model for estimating and evaluating the WQI. Their work revealed significant potential for nonlinear models in water quality assessment. Models utilizing a spectral index of 1.6 outperformed the others, achieving R^2^ of 0.92, RMSE of 58.4, a curve-fitting slope of 0.97, and RPD of 2.81.

**Figure 4:**
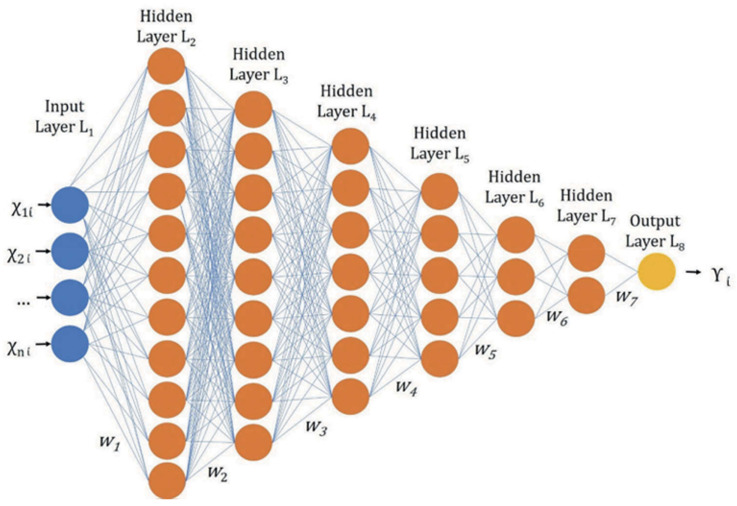
An illustration of the progressively decreasing deep neural network structure [16].

Parameters like specific conductance (SC) and dissolved oxygen (DO) lack optical activity, posing a unique difficulty for remote sensing [31]. In addressing this core problem, an indirect estimate of DO and SC using other proxy variables presents a practical approach. In developing an empirical algorithm to determine DO from satellite data, both satellite-derived data and on-site observed DO satellite-derived data (SST and Chl-a concentrations) were employed in a stepwise multiple regression analysis [78]. Their findings revealed a robust inverse relationship between DO and water temperature, aligned with principles of gas solubility. In this work, the researchers developed a multiple regression model to estimate DO in surface waters. The model was based on correlating on-site DO measurements with satellite-derived Chl-a level measurements and water temperature. Consequently, the satellite-derived DO algorithm enables the detection of sustained variations in DO concentration. To enhance model performance, researchers are increasingly exploring advanced empirical methods, including neural networks [79], and physics-based inversion methods. Some neural network-based approaches [80] have demonstrated notable success with MERIS data, with applicability to lakes and water bodies sharing similar optical properties. Despite extensive efforts in water quality variable estimation, achieving high accuracy remains elusive, often limited to specific sites or single bodies of water. While machine learning shows promise in overcoming these challenges, the intricate connections among solar radiation and water constituents pose complexities for traditional machine learning approaches [16]. However, deep learning techniques, which capture higher-order statistical relations, frequently outperform conventional calculation and modeling techniques. Given the specific difficulties inherent in water quality modeling, careful consideration must be given to model development [16]. Figure 4 illustrates the progressively decreasing deep neural network structure. In this regard, C. Niu et al. [72] showcased that deep learning-driven regression models exhibit strong proficiency in extracting features and understanding images from high-dimensional data, thus offering a novel avenue for estimating optically inactive parameters in inland water quality. The study compared the accuracy of deep neural network regression (DNNR) methods with the traditional regression methods including partial least squares regression (PLSR) and support vector regression (SVR). It estimated the organic pollution parameters COD_Mn_, NH3–N, TN, and TP, as well as the heavy metals Zn, Cd, and Ni. The PLSR model demonstrated the poorest accuracy, with the coefficient of determination (Rp^2^) values remaining below 0.6, with the exception of TN. The patch-based DNNR model obtained exceptional prediction accuracy across all seven water quality parameters, achieving (Rp^2^) values exceeding 0.6 and residual prediction deviation (RPD) values above 1.6 for the prediction dataset. This indicates superior regression performance.

As stated by H. Yang et al. [35], water quality parameters can be obtained through four distinct approaches: empirical, analytical, semi-empirical, and artificial intelligence mode.

- The empirical mode relies on statistical correlations between measured remote sensing (RS) spectral values and observed water quality parameters, determined through regression techniques. Nevertheless, empirical models necessitate on-site data for estimations due to potential parameter variations between RS missions, offering simplicity in water quality information retrieval [35].
- Bio-optical and radiation transmission models are employed in the analytical mode. These models simulate light transmission in water bodies and the atmosphere. The aim is to correlate water quality components with off-water radiation spectra. However, complexities in water composition and radiation transmission processes pose challenges, especially given inconsistencies in spectral resolution between ground measurements and satellite sensors, limiting practical applications [50].
- Semi-empirical methods blend empirical and analytical approaches by leveraging knowledge of parameter spectral characteristics and selecting appropriate waveband combinations as correlates. These methods recalibrate spectral radiance into above-surface irradiance reflectance, employing regression techniques to link with water quality parameters [35,50].
- The artificial intelligence mode (AIM) employs implicit algorithms distinct from the other modes, catering to the complexities of diverse water surfaces, water quality parameter combinations, and sediment deposits. AI applications excel in capturing both linear and nonlinear relationships, offering promising outcomes in water quality retrieval. Various AI techniques, including neural networks (NN), outperform conventional statistical approaches like support vector machines and multiple linear regression, contributing to enhanced water quality estimation [35,81].

Table 5 outlines a detailed summary of the various sensors employed by researchers in advancing remote sensing techniques for monitoring water quality. Additionally, it presents the list of algorithms or methodologies they devised to identify water quality parameters. It also includes critical performance metrics such as the coefficient of determination (R^2^), which indicates the goodness of fit between observed and predicted values, and the root mean square error (RMSE), which reflects the model’s prediction accuracy. By presenting these datasets and algorithms alongside their associated accuracy parameters, the table comprehensively compares their effectiveness for water quality parameters estimation.

Analysis of the data presented in Table 5 reveals that researchers primarily adopted two approaches for modeling water quality using remote sensing data. Empirical modeling, hinges solely on statistical methods, whereas semi-analytical (bio-optical), is grounded in the physics governing light interaction with water surfaces. Empirical techniques aim to establish connections among water quality constituents and spectral reflectance values (either individual spectral bands or their combinations) through regression-based analysis. These approaches are data-driven and require on-site water quality measurements to develop empirical relationships, often using linear or non-linear regression, among water quality metrics and the sensor-measured water-leaving radiance. Given the optical complexity of freshwater bodies, most empirical methods adopt a multivariate regression modeling approach. In contrast, bio-optical modeling, considered analytical, centers on radiative transfer inside the water column. By employing the radiative transfer equation, these models derive optically active elements from water-leaving radiance, necessitating detailed spectral data of these constituents within the target area. Rooted in the interactions between light and water, these approaches seek to address and overcome the challenges of regional transferability that are inherent in empirical techniques [58]. Notably, Laili N. et al. [99] developed an algorithm that demonstrated a strong correlation between in situ measurements and remote sensing-derived estimates of Chl-a and TSS concentrations across nine stations as shown in Table 6.

Remote sensing of aquatic color radiometry in coastal and inland water bodies is of significant interest to researchers, management agencies, commercial sectors, and the general public. However, the majority of existing satellite radiometers were initially designed for global ocean observation, which makes their use in coastal and inland waters more challenging. Nonetheless, substantial progress has been made in advancing in situ observations, boosting operational capabilities, strengthening user engagement, and developing algorithms [115,116,117]. A key issue in satellite measurements of coastal and inland waters is the presence of land adjacency effects (LAE) near land-water boundaries [118]. To mitigate this, a statistical method was introduced to quantify LAE in the short-wave infrared signals from MODIS Aqua, leading to the development of a Look-Up Table to guide the correction of these effects. While initial progress has been made in reducing the number of invalid pixels through this correction, further refinement is needed to improve its overall effectiveness.

Furthermore, the accuracy of surface reflectance data are insufficient for use in various inversion models designed to estimate essential water quality parameters, including water clarity, turbidity, TSS, and Chl-a concentration. Hence, proper atmospheric corrections are essential before conducting such analyses [50,119,120]. The primary challenge lies in the complexities of performing these corrections over optically complex waters. Feng L. et al. [121] addressed this challenge by demonstrating that although MODIS Aqua surface reflectance data products (R_Land) were originally designed for land applications, they can be effectively utilized for water environments, particularly in inland and estuarine regions. The study found that R_Land(645) and R_Land(645/555) provide high accuracy when compared with in situ measurements and reflectance products obtained using water-specific atmospheric correction methods (R_NIR based on near-infrared bands and R_SWIR based on shortwave-infrared bands). Additionally, the study showed that data quality can be enhanced through spatial and temporal binning. Given the limitations users face in generating custom R_SWIR data and the often insufficient coverage of NASA’s standard R_NIR products for inland waters, this research offers valuable guidance on the applicability and effectiveness of the widely available R_Land products for such water bodies.

### Recent Advancement

The application of IoT sensors for remote sensing in water quality monitoring is an exciting and rapidly evolving field, where IoT technologies enable smart sensors to transform how we assess and manage water resources. Through the use of IoT, these sensors are interconnected via wireless networks, allowing them to collect, process, and transmit data from even the most remote locations in real-time [122]. In this context, Prasad et al. [123] proposed a smart water quality monitoring system that leverages these capabilities. The water quality parameters analyzed include Oxidation–Reduction Potential (ORP) and pH. The successful implementation of this monitoring approach will lead to the establishment of an early warning system for water pollution, supported by a fully functional network of multiple monitoring stations.

Jerom B. et al. [124] introduced a Smart Water Quality Monitoring System that utilizes IoT, Cloud, and Deep Learning techniques to monitor the water quality of different water resources. The developed system enables continuous water quality monitoring through the use of IoT devices and a Node-MCU. The integrated Wi-Fi module in the Node-MCU ensures internet connectivity and transmits the sensor data to the Cloud for further analysis. The designed prototype monitors several contaminants in the water using various sensors to measure different parameters for assessing water quality in water resources. The collected data are stored in the Cloud, where deep learning techniques are applied to predict whether the water is potable or not.

The research on smart sensors for remote sensing applications is still developing, and while there has been significant progress, the body of literature remains relatively limited. This limitation can be attributed to several factors, including technological, methodological, and practical challenges in deploying and utilizing smart sensors in remote sensing applications. However, the increasing focus on environmental monitoring, climate change, and sustainable development is expected to drive further advancements and innovation in smart sensor technology, opening the door for more comprehensive and widespread applications in the future.

## 4. Discussion

Remote sensing encompasses the collection, processing, and analysis of images and associated data, usually obtained from aircraft and satellites equipped with sensing technology that digitally captures the interaction among electromagnetic energy and substances. This interaction is influenced by the physical characteristics of the substances and the wavelength of the electromagnetic energy being sensed remotely. Electromagnetic energy can be characterized by its speed, wavelength, and frequency. Table 7 provides an overview of the electromagnetic spectrum and its applications related to remote sensing.

Remote sensing images of the Earth provide numerous practical applications, particularly in water monitoring and resource management, where they play a crucial role in assessing and managing water quality and availability. Traditionally, assessing water quality parameters involves collecting field samples and analyzing in a laboratory. Though this process is accurate, it is time-consuming and demanding. This makes it impractical to develop a complete regional water quality database in a single effort. Furthermore, conventional point sampling methods struggle to capture geographical and time-related fluctuations in water quality. Capturing these fluctuations is crucial for a comprehensive assessment and management of water bodies. Consequently, these challenges in sequential and integrated sampling pose a substantial barrier to tracking and managing water quality. The key constraints of traditional approaches are outlined below [125].

- Sampling and measurement of water quality indicators with in-suit methods are laborious, time-consuming, and expensive.
- Examining the temporal and spatial fluctuations and trends in water quality within expansive water environments is nearly unattainable.
- The monitoring, prediction, and management of whole aquatic systems may be unfeasible, particularly due to topographic constraints.
- Reliability and specificity of in situ data collected may be uncertain, influenced by both laboratory errors and field sampling.

Remote sensing can be a valuable resource for overcoming these constraints in water quality assessment. Additionally, advancements in space technology and the growing utilization of software applications have contributed to the development of remote sensing techniques. Improved computing power over the past few years has also played a significant role. Consequently, remote sensing techniques have become invaluable tools for this purpose. These methods enable more powerful and streamlined monitoring and identification of extensive areas and water bodies facing quality issues. The data collected through remote sensing is in digital format, facilitating easy interpretation and processing by computers. Below are the primary benefits of utilizing remote sensing in water monitoring:

- Offers a comprehensive view of the entire water body, enhancing the monitoring of temporal and spatial changes effectively.
- Enables synchronized water quality assessment across a group of lakes spanning an extensive region.
- Offers a detailed historical water quality record in a specific area, depicting patterns over time.
- Assists in optimizing the selection of sampling sites and scheduling field surveys.

**Figure 5:**
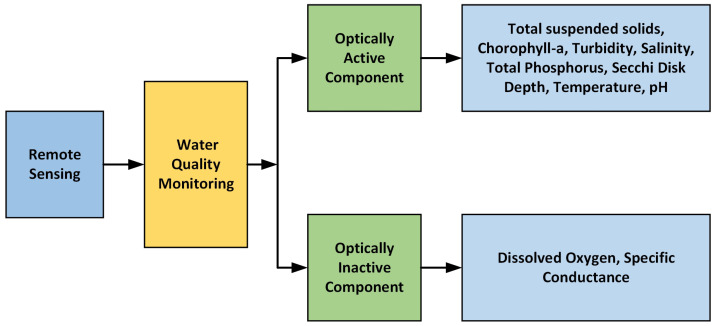
Block diagram representation of remote sensing applications in the field of water quality monitoring.

Remote sensing is widely used in water monitoring to assess quality by detecting indicators that are classified as either optically active or optically inactive constituents. Optically active constituents include factors such as turbidity, salinity, transparency, Chl-a concentration, total suspended solids (TSS), total phosphorus, pH, and temperature. In contrast, dissolved oxygen and specific conductance are considered optically inactive constituents (Figure 5).

Observational sensors are divided into two primary groups according to their deployment platforms. The sensors installed on airborne platforms within Earth’s airspace, such as balloons, boats, aircraft, or helicopters, are known as Airborne sensors, while space-borne sensors are transported by spacecraft or satellites to sites beyond Earth’s airspace. Both airborne and satellite remote sensing methods are valuable for analyzing freshwater quality. Airborne sensors present greater flexibility compared to space-borne counterparts due to their superior spatial and spectral resolution, as well as a wider range of spectral bandwidth. This facilitates more accurate retrieval of water quality parameters. Airborne sensors excel in observing smaller water bodies like effluents, rivers, small lakes, and river mouths, whereas satellite sensors are better suited for observing bigger water bodies. Space-based sensors are pivotal in understanding and managing Earth’s water resources. Installed on satellites orbiting the planet, these sensors feature sophisticated instruments capable of capturing various water quality parameters. They rely on detecting and analyzing electromagnetic radiation emitted or reflected by water bodies to gather data on factors like temperature, turbidity, chlorophyll concentration, and pollution levels. Space-based sensors offer the benefit of delivering an extensive overview of water quality across large geographical regions. By capturing data from a bird’s-eye perspective, these sensors offer a broader understanding of water quality trends and variations across different regions. Scientists and decision-makers can make informed choices about water resource management and conservation based on this comprehensive perspective. Space-borne sensors generally encompass larger geographic regions compared to airborne counterparts, but they tend to exhibit comparatively lower spectral and spatial resolutions [33]. In water quality monitoring, when selecting sensors for remote sensing methods, the main considerations include area of coverage, spectral resolution, and spatial resolution.

Space-borne and airborne sensors are categorized into multispectral and hyperspectral types based on the number and width of their spectral bands. The primary distinction lies in their spectral capture capabilities. Multispectral sensors typically acquire a limited number of spectral bands (usually between 3 and 10) with broader bandwidths, concentrating on specific regions of the spectrum, such as the visible and near-infrared ranges. In contrast, hyperspectral sensors capture hundreds of narrow spectral bands, facilitating more detailed spectral analysis and enabling precise material identification. However, achieving such high spectral resolution requires advanced computational capabilities, complex data processing, and specialized expertise for accurate interpretation of the results. On the other hand, high-resolution multispectral satellite images provide detailed visualizations of land cover. When a bird’s-eye view is sufficient and ultra-fine spectral discrimination is not required, multispectral imaging emerges as a cost-effective solution that efficiently fulfills project needs. Ultimately, when deciding between multispectral and hyperspectral imaging, it is essential to evaluate which factor is more critical for the task: the need for higher spectral detail or the importance of operational efficiency.

Researchers have utilized information from various satellites, including Terra, EO1 Sentinel-2, Landsat, and Envisat, to determine correlations between spectral reflectance and water quality parameters. Wavelengths of 675 nm and 700 nm have been identified as the most effective for chlorophyll detection, while the red and near-infrared wavelengths are advantageous for assessing turbidity and TSS. Furthermore, using band ratios to integrate reflectance from multiple bands has been found to enhance water quality parameter estimates through the reduction of atmospheric effects and the improvement of signal quality. The SSD demonstrates an inverse relationship with turbidity and TSS concentration, thus its estimation relies on observed correlations with TSS and turbidity. Likewise, CDOM is strongly correlated with Chl-a, TSS, and turbidity levels. The majority of developed algorithms are empirically based, necessitating precise parameterization that can differ depending on the optical properties of the water system. In recent years, there have been efforts to integrate hyperspectral sensor data alongside multispectral data. This integration seeks to enhance the precision of estimating water quality parameters.

### Challenges Related to Remote Sensing in Water Monitoring

Remote sensing proves to be a fitting method for examining the spatial and temporal fluctuations of water quality variables. Nonetheless, several significant constraints warrant careful consideration before employing this method. Remote sensing models require thorough calibration and validation using in situ data for accurate results. Additionally, passive sensors operating in the solar reflective spectrum are limited to cloud-free conditions, restricting their applicability when cloud cover is present. Furthermore, in certain scenarios, the accuracy of extracted water quality parameters might be open to debate [18]. For example, Kutser [126] argues that standard satellite products frequently fail to identify the densest regions of cyanobacteria blooms in the Baltic Sea caused by atmospheric corrections or data processing inaccuracies.

Numerous existing optical sensors can potentially limit the use of remotely sensed data for assessing water quality due to spatial, temporal, and spectral resolution constraints. Moreover, crucial parameters such as water discharge and the vertical distribution of water quality metrics within water bodies are difficult to estimate directly with optical sensors. The primary challenge of using remote sensing techniques for water quality monitoring is the expense associated with airborne or hyperspectral data and the necessary instrumentation for conducting in situ hyperspectral measurements. Additionally, optical challenges in interior and shoreline waters further limit remote sensing applicability [18].

The disparity in sampling volumes or scales between in situ measurements and sensor data presents a significant challenge in remote sensing and environmental monitoring. This scale mismatch complicates the integration of in situ data with remote sensing observations and can introduce errors and uncertainties in modeling and analysis. A critical issue is that the relationships between satellite measurements and in situ ocean properties are often not well understood. Another challenge is the lack of accurate surface reflectance data to be used as inputs of the various inversion models to estimate water quality parameters such as turbidity, water clarity, TSS, and Chl-a concentration, among others [127,128]. Additionally, measurements from different satellite sensors may not be directly comparable due to variations in sensor characteristics. The scale disparity between satellite and in situ measurements creates challenges for machine learning (ML) and artificial intelligence (AI) algorithms. These challenges include difficulties in data representation, integration, accuracy, and overall model performance. To address these issues, careful data preprocessing, feature engineering, and model selection are essential to ensure that ML algorithms can effectively learn from and make accurate predictions based on satellite-to-in situ matching pairs.

To tackle these challenges, Kahru et al. [127] developed satellite algorithms empirically tuned to match in situ datasets of inherent optical properties, aiming to minimize sensor-to-sensor differences. Furthermore, Feng et al. [121] proposed quality-control criteria for selecting satellite-to-in situ matching pairs, emphasizing the exclusion of satellite pixels with a high coefficient of variation to enhance data accuracy and reliability. Effective use of remote sensing data for applications such as water quality monitoring requires meticulous calibration with in situ data. Addressing disparities in sampling volumes involves sophisticated statistical and analytical methods to ensure that remote sensing observations accurately reflect the conditions measured in situ.

The documentation regarding the separation of spectral characteristics for inorganic suspended matter, CDOM, and Chl-a is insufficient in existing research, posing a challenge due to the mutual influence of these parameters. Additionally, atmospheric interference limits the optical measurements received from water bodies. In pristine ocean environments, the expected maximum light penetration depth is approximately 55 m around 475 nm, with most incident energy being absorbed and/or transmitted at the water surface. However, as the concentration of suspended sediment increases to, for instance, 400 mg/L, the penetration depth diminishes to just 60 cm. Consequently, only a gradually shallower layer of surface water becomes observable [18].

**Figure 6:**
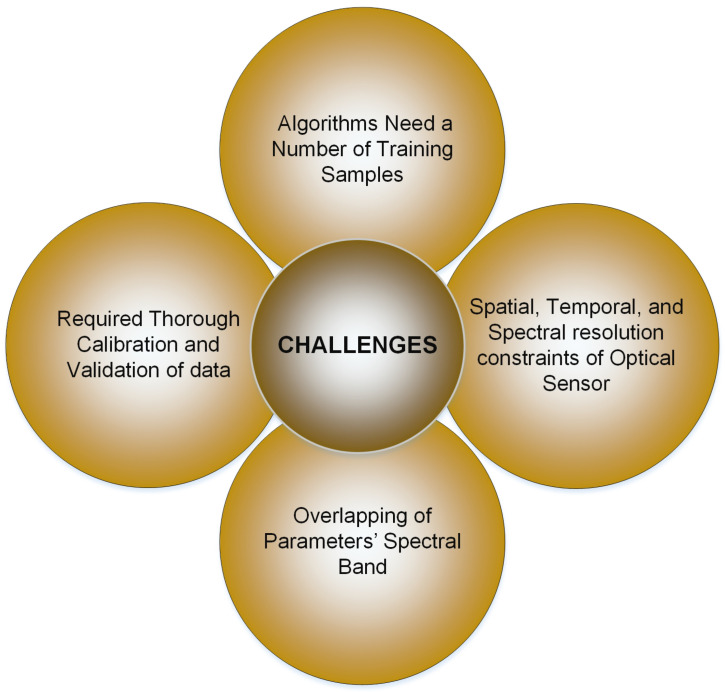
Challenges related to remote sensing techniques in water monitoring.

Various algorithms possess distinct strengths and necessitate varying input parameters. Numerous variables, such as study area scale, software availability, spatial resolution of remotely sensed data, and analyst knowledge and skills, influence the modeling method. Calibration and validation of the developed model utilizing in situ measurements are crucial for remote sensing data and are possible only when there are no clouds present. Machine learning algorithms rely on a substantial quantity of training samples, which are difficult to obtain in practical situations [1]. The comprehensive challenges related to remote sensing techniques in water monitoring are depicted in Figure 6.

## 5. Conclusions

Despite being crucial, freshwater resources are facing growing challenges to their water quality as a result of factors like demographic increase, human activities, global warming, and various anthropogenic pressures. Remote sensing methodologies offer a means to monitor various water quality parameters with acceptable accuracy (see Table 6), provided that appropriate algorithms, calibration, and validation procedures are implemented. Thermal and optical sensors installed on airplanes, satellites, and boats provide location-based and time-series data. These data are critical for monitoring variations in water quality and developing operational policies to enhance water quality. The launch of satellites equipped with advanced spectral and spatial resolution sensors such as Sentinel-2, Landsat, and MODIS, along with upcoming missions, is expected to significantly enhance the application of remote sensing techniques for assessing and monitoring water quality parameters. The combination of GPS and GIS technologies, along with remotely sensed data, provides an efficient resource for continual monitoring and evaluation of water bodies. Utilizing remote sensing data enables the creation of a durable geographically referenced database, which can serve as a reference for future assessments.

In evaluating the quality of freshwater bodies, satellite and airborne remote sensing methods prove valuable tools. Satellite sensors excel at observing larger water bodies, while airborne sensors are more effective for monitoring smaller bodies of water, such as creeks, basins, and tidal mouths. Utilizing multiple satellite images can help evaluate water quality. Empirical methods are simple to apply and demand minimal mathematical knowledge and computational effort. However, these techniques may be ineffective for metrics lacking distinct absorption features, such as DO, and to some extent, suspended matters. Analytical approaches can determine all water constituents simultaneously, provided that the essential traits of indicators are clearly understood and a large quantity of on-site data are available. Most studies have focused on light-sensitive parameters like Chl-a, CDOM, turbidity, and TSS. Nevertheless, certain crucial water quality parameters, such as pH, dissolved phosphorus, ammonia nitrogen, nitrate nitrogen, and total nitrogen, remain unaddressed due to their faint photosensitive properties and high noise levels. Although these constraints are well acknowledged, remote sensing remains a valuable resource for monitoring water quality. In this context, water quality monitoring is closely linked to environmental science and ecology, with remote sensing playing a pivotal role in assessing and managing water quality. By providing large-scale, real-time data on aquatic ecosystems, remote sensing is essential for understanding the health of water bodies, their impact on surrounding environments, and their role in broader ecological systems. Specifically, water quality is deeply connected to the health of wetlands and coastal ecosystems, which are highly sensitive to pollution and climate change. Remote sensing enables continuous monitoring of critical water quality parameters, such as salinity, temperature, and chlorophyll concentrations, which directly affect the health of these ecosystems and their biodiversity.

In conclusion, while challenges persist in monitoring certain water quality parameters, the integration of remote sensing with environmental science and ecology offers an indispensable tool for managing water resources and preserving ecosystem health. The continuous advancements in remote sensing technologies hold the potential to overcome current limitations, further enhancing our ability to monitor and protect aquatic environments sustainably.

## Figures and Tables

**Table 1:** Comparison of multispectral and hyperspectral sensors.

Feature	Multispectral Sensors	Hyperspectral Sensors
Spectral Bands	3–10 bands	100+ bands
Spectral Resolution	Low (broad bands)	High (narrow bands)
Spatial Resolution	Typically higher	Typically lower
		(due to large number of bands)
Data Volume	Lower volume	Higher volume
Data Processing	Simpler	More complex
		(requires advanced algorithms)
Cost	Lower, widely available	Higher, specialized missions
Applications	General land cover, agriculture,	Specific detection
	water quality	(minerals, pollutants, plant stress)

**Table 2:** Specification of space-borne sensors frequently used in water monitoring [18].

Category	Satellite-	Spectral	Spectral	Swath	Temporal
	Sensor	Bands	Resolution	Width	Resolution
		(nm)	(m)	(km)	(Days)
High	Digital Globe	Pan	0.5	17.7	1.7
Resolution	WorldView-1				
	Digital Globe	8 (400–1040)-1	1.85–0.46	16.4	1.1
	WorldView-2	Pan (450–800)			
	NOAA	8 (400–1040)-1	1.24–3.7	13.1	1–4.5
	WorldView-3	Pan (450–800)-8	−0.31		
		SWIR (1195–2365)			
	Quickbird	Pan (450–900)			
	Digital Globe	4 (430–918)-1	2.62–0.65	18	2.5
	GeoEye	4 (450–920)-1	1.65–0.41	15.2	<3
		Pan (450–800)			
	GeoEye	4 (445–853)-1	3.2–0.82	11.3	∼3
	IKONOS	Pan (526–929)			
	CARTOSAT	Pan (500–850)	2.5	30	5
Moderate	Landsat-8	5 (430–880)-1	30–15–100	170	16
Resolution	OLI/TIRS	Pan (500–680)-2			
		SWIR (1570–2290)-1			
		cirrus cloud detection			
		(1360–1380)-2			
		TIRS (10,600–12,510)			
	TM	1 (2080–2350)-			
		1 (1040–1250)			
	EO-1	242 (350–2570)	30	7.5	16
	Landsat-7	6 (450–1750)-1	30–15–60	183	16
	Landsat-5	4 (450–1750)-	80	185	18
	Landsat-5	5 (450–1750)-	30–120	185	16
	ETM+	Pan (520–900)-			
		1 (2090–2350)-			
		1 (1040–1250)			
	MSS	1 Pan (1040–1250)			
	Terra	3 VNIR (520–860)-	15–30–90	60	16
	Hyperion				
	EO-1 ALI	9 (433–2350)-	10–30	185	16
		1 Pan (480–690)			
	ASTER	6 SWIR (1600–2430)-			
		5 TIR (8125–11,650)			
	HICO	128 (350–1080)	100	45–50	10
	PROBA	19 in the VNIR	18–36	14	7
	CHRIS	range (400–1050)			
Regional-	Terra	2 (620–876)-	250–500–1000	2330	1–2
Global	MODIS	5 (459–2155)-			
Resolution		29 (405–877			
		and thermal)			
	OrbView-2	8 (402–885)	1130	2806	16
	SeaWiFS				
	Envisat-1	15 (390–1040)	300–1200	1150	daily
	MERIS				
	NIMBUS-7	6 (433–12,500)	825	1556	6
	CZCS				
	ATSR-2	1 SWIR (1600),			
		1 MWIR (3700),			
		2 TIR (10,850–12,000)			
	ERS-2	3 VIS-NIR (555–865),	1000	500	3–6
	ERS-1	1 SWIR (1600),	1000	500	3–6
	ATSR-1	1 MWIR (3700),	(MW sounder:		
		2 TIR (10,850–12,000),	20 km)		
		Nadir-viewing			
		Microwave Sounder with			
		channels at 23.8			
		and 35.6 GHz			
	AATSR	1 SWIR (1600),			
		1 MWIR (3700),			
		TIR (10,850–12,000)			
	ENVISAT	3 VIS-NIR (555–865),	1000	500	3–6
	Suomi	5 I-bands (640–1145),	375–750	3060	1–2
	NOAA-16	6 (650–1230)	1100–4000	3000	9
	AVHRR				
	NPP VIIRS	16 M-bands			times
		(412–12,013),			a day
		DNB (500–900)			

**Table 3:** Specifications of commonly used air-space sensors in water quality monitoring [35].

Sensors Types	Full Name	Type	Scan Scheme	Band Count	Spectral Range	Resolution	Imaging Swath
					(μm)	(m)	
AISA	Airborne Imaging	Hyperspectral	Pushbroom	Up to 288	0.43–0.90	1	364
	Spectrometer						pps
APEX	Airborne Prism Experiment	Hyperspectral	Pushbroom	Up to 300	VIS/NIR (0.38–0.97),	2 to 5	2.5–5 km
				VIS/NIR (114),	SWIR1 (0.97–2.50)		
				SWIR (199)			
AVIRIS	Airborne Visible Infrared	Hyperspectral	Whiskbroom	224	0.40–2.50	17	12 km and
	Imaging Spectrometer						614 pps
CASI-1500	Compact Airborne	Hyperspectral	Pushbroom	Up to 228	0.40–1.00	0.5 to 3	512 pps
	Spectrographic Imager						
DAIS 7915	Digital Airborne	Hyperspectral	Whiskbroom	VIS/NIR (32),	VIS/NIR (0.43–1.05),	3 to 20	512 pps
	Imaging Spectrometer			SWIR1 (8),	SWIR1 (1.50–1.80),	dependent on	
				SWIR2 (32),	SWIR2 (2.00–2.50),	altitude	
				MIR (1),	MIR (3.00–5.00),		
				TIR (12)	TIR (8.70–12.30)		
Daedalus	Daedalus Multispectral	Multispectral	Pushbroom	12 VIS/NIR (8),	0.42–14.00	25	714 pps
	Scanner (MSS)			SWIR (2),			
				TIR (2)			
EPS-H	Environmental	Hyperspectral	Whiskbroom	VIS/NIR (76),	VIS/NIR (0.43–1.05),	Depending	89°
	Protection System			SWIR1 (32),	SWIR1 (1.50–1.80),	on flight	
				SWIR2 (32),	SWIR2 (2.00–2.50),	(min 1 m)	
				TIR (12)	TIR (8–12.50)		
HYDICE	Hyperspectral Digital	Hyperspectral	Pushbroom	210	0.40–2.50	0.8 to 4	270 m at
	Imagery Collection Experiment						lowest altitude
HyMap	in the U.S. known	Hyperspectral	Whiskbroom	128	0.40–2.50	3 to 10	512 pixels
	as PROBE-1						
HySpex	HySpex	Hyperspectral	Pushbroom	VIS/NIR1 (128),	0.40–2.50	0.5 at	500 m
MIVIS	Multispectral Infrared	Multispectral	Whiskbroom	102 VIS/NIR (28),	VIS (0.43–0.83),	3 to 8	5.6 km at
	and Visible Imaging			MIR (64),TIR (10)	NIR (1.15–1.55),	depending on	4000 m
	Spectrometer				MIR (2.0–2.5)	altitude	altitude
					TIR (8.2–12.7)		
ODIN-1024	hyperspectral			VIS/NIR2 (160),		2000	
	cameras			SWIR1 (160), SWIR2 (256)		altitude	

pps: pixel per scanline.

**Table 4:** Contrast between satellite-based and airborne sensors [33].

Criterion	Satellite-Based (Space-Borne)	Airborne
Temporal resolution	In days	In minutes
Spectral resolution	Primarily ranging from panchromatic	Panchromatic to
	(single-band) to multispectral,	hyperspectral
	newly designed sensors such as CHRIS,	
	HICO, and HyspIRI exhibit hyperspectral	
	capabilities	
Spatial resolution	The Ground Sampling Distance (GSD)	The Ground Sampling
	for panchromatic images can	Distance < 5 m
	be up to 0.5 m. In the case of	
	multi-band images, the GSD varies from	
	a few meters to several kilometers	
	(from low-altitude sensors to	
	high-altitude sensors).	
Calibration	Pre-calibration prior to launch followed	Prior to launch and
	by on-board characterization	likely on-board
	(typically annual)	
Time of overpass	Mostly fixed	Flexible
Stability	High	Low (caused by turbulence)
Expense	Free for non-commercial, up to about	Typical expenses are
	USD 50/km^2^	USD 350/mile^2^
	High spatial resolution imagery are	
	quite costly (∼$2000–10,000	
	per scene)	
Swath width	High (covering an entire hemisphere	Small (as much as 10 km
	for high-altitude sensors, as much as	per flight time)
	2500 km for low-altitude sensors)	
Image processing	Less complex	Processing of hyper-
complication		spectral images is
		more intricate and
		demands specialized skill set
Constraints	Constrained by the satellite’s	The coverage timetable
	coverage timetable, which encompasses	can be adjusted
	cloud and weather conditions, conducting	as needed.
	water quality monitoring during particular	
	periods or adhering to project	
	timelines can present challenges.	
Geographic coverage	Global, regional, and local	Local and regional
areas		

**Table 5:** Different sensors used to detect water quality parameters through remote sensing.

Satellite/	Sensor Used	R^2^	RMSE	Method/Algorithm	Ref.
Other Platform					
Chl-a					
Landsat-5	TM	0.83	1.13	Exponential Regression	[82]
			mg/m^3^		
Landsat-7	ETM+	0.723	-	Empirical	[83]
		0.67		Regression Model	[84]
		-		Support Vector Regression	[85]
		0.89		Genetic Algorithm	[86]
Landsat-5 and 7	TM and ETM+	0.89		Deming Regression	[87]
		0.72		Linear mixed model	[88]
Landsat 7	ETM+	0.76	13 μg/L	Multiple Linear Regression	[89]
Landsat 8	OLI	0.89	5.1%	3 BDA	[4]
Landsat 8	OLI	0.774	22.636	Support Vector Machine	[90]
			μg/L		
Envisat	MERIS	-		Neural network	[91]
		0.76		Semi-analytical model-	[92]
Sentinel-2	-	>0.87	6 μg/L	Multiple Linear Regression	[93]
Sentinel-2	-	0.8	28%	3 BDA	[94]
Terra	MODIS	0.86		Empirical Regression	[95]
				Optimizing and	
				look-up-table	
Terra and Envisat	MODIS and MERIS	>0.98			[96]
Airborne	hyperspectral	-		BOMBER inversion	[97]
		-		Generalized Inherent	[98]
				Optical Property	
TSS					
Landsat 8	OLI	0.709	0.0967	Regression model	[99]
Landsat 8	OLI	>0.9	<10 %	Empirical Regression model	[100]
Sentinel-2	MSI	between	<10 %	Empirical Regression model	[100]
		0.47&			
		0.61			
CDOM					
Landsat 4, 5 and 7	TM and ETM+	0.62		Empirically based	[101]
Landsat 5 and 7	TM and ETM+	0.78		Empirically based	[102]
Landsat 8 and	OLI and	-		Peak height	[103]
Sentinel 2	MSI				
Landsat 5	TM	-		-	[104]
Envisat	MERIS	0.79		Empirically based	[105]
Envisat	MERIS	-		Neural network	[91]
EO-1	ALI	-		Empirically based	[106]
Landsat 8	OLI	0.87		Shallow water Bio-Optical	[107]
				Optical Property algorithm	
Satlantic	HyperSAS	0.74		Shallow water Bio-Optical	[108]
				Optical Property algorithm	
Airborne	hyperspectral	-		Analytical Modular	[109]
				inversion and processing	
				system	
Dissolved Oxygen					
Landsat 4, 5 and 7	TM and ETM+	0.91		Support Vector Regression	[110]
Landsat 7	ETM+	0.81		Multiple Regression Models	[111]
Landsat 8 and	OLI and	0.89		Principal Component Analysis	[112]
Sentinel 2A	MSI			based Response Surface	
				Regression	
Terra and Envisat	MODIS and MERIS	0.801		Multiple Regression	[78]
Metop	-	0.32		Support Vector Regression	[113]
SPOT-5	-	0.97		Support Vector Regression	[114]

**Table 6:** Estimated and measured values of TSS and Chl-a.

Station	TSS (g/m^3^)		Chl-a (mg/m^3^)	
	Estimated	Measured	Estimated	Measured
ST1	12.526	14	248.8729	278
ST2	13.294	13	263.228	286
ST3	9.480	13	238.546	298
ST4	14.464	15	295.538	280
ST5	12.769	14	252.5938	254
ST6	14.779	16	306.2124	386
ST7	15.801	18	346.6274	459
ST8	15.363	17	328.1409	327
ST9	15.152	16	319.9305	332

**Table 7:** Application of electromagnetic spectrum in remote sensing [34].

Spectral Band	Wavelength	Application
Gamma ray region	<0.03 nm	Not used in remote sensing
X-ray region	0.03 to 30 nm	Not used in remote sensing
UV region	0.03 to 0.4 μm	The ozone layer absorbs incident radiation
		with wavelengths shorter than 0.3 μm
Far UV band	0.3 to 0.4 μm	Transmitted through atmosphere,
		but scattering is severe
Visible region	0.4 to 0.7 μm	
blue band	0.4 to 0.5 μm	Absorbed by chlorophyll in plants
		Scattered by atmosphere.
green band	0.5 to 0.6 μm	Includes maximum reflected
		energy from earth
red band	0.6 to 0.7 μm	Absorbed by chlorophyll in plants
Infrared Region	0.7 to 1000 μm	
NIR range	0.7 to 0.9 μm	Solar radiation that is highly
		reflected by vegetation
SWIR range	0.9 to 3.0 μm	SWIR spectra are used
		to identify minerals
TIR range	3.0 to 5.0 μm	suitable for “hot” targets
		(e.g., fires and volcanoes)
	8.0 to 14.0 μm	suitable for “warm” targets
		(e.g., territory and waters)
Microwave region	0.1 to 100 cm	
Passive methods	0.1 to 100 cm	Used for soil moisture
		and other investigation
Active methods	0.8 to 100 cm	Different wavelength bands are
		utilized for wide range of applications
Radio region	>100 cm	Primarily used in communication
